# Digital health literacy and disordered eating among adolescents: the chain mediating roles of physical activity and problematic social media use

**DOI:** 10.3389/fpsyt.2026.1937781

**Published:** 2026-08-19

**Authors:** Xuyao Ding, Chuyuan Zhao, Jiaqi Qu, Chen Tang, Chuxi Shang, Jianing Lyu, Zhen Li

**Affiliations:** 1 College of Physical Education, Henan University, Kaifeng, Henan, China; 2 Body-Brain-Mind Laboratory, School of Psychology, Wuhan Sports University, Wuhan, China; 3 School of Physical Education, Henan Kaifeng College of Science Technology and Communication, Kaifeng, Henan, China

**Keywords:** adolescents, digital health literacy, disordered eating, physical activity, problematic social media use

## Abstract

**Background:**

Digital environments may increase adolescents’ vulnerability to disordered eating through misleading health information and problematic social media use. This study examined the association between digital health literacy and disordered eating, including the independent and serial indirect associations of physical activity and problematic social media use.

**Methods:**

This cross-sectional study included 2,206 adolescents recruited from five secondary schools using purposive and convenience sampling. Participants had a mean age of 14.50 years (SD = 1.76). Digital health literacy, disordered eating, physical activity, and problematic social media use were assessed using validated scales. Correlation and bootstrap-based regression analyses were conducted to estimate the specific and serial indirect associations.

**Results:**

Digital health literacy was negatively associated with disordered eating and problematic social media use and positively associated with physical activity. The total association between digital health literacy and disordered eating was −0.324 (95% CI [−0.365, −0.283]), including a direct association of −0.135 (95% CI [−0.177, −0.092]). Indirect associations were observed through physical activity (−0.098, 95% CI [−0.121, −0.077]), problematic social media use (−0.072, 95% CI [−0.089, −0.057]), and physical activity followed by problematic social media use (−0.019, 95% CI [−0.024, −0.015]).

**Conclusion:**

Digital health literacy was associated with disordered eating through independent and serial indirect associations involving physical activity and problematic social media use. These findings suggest the value of coordinated school- and family-based strategies integrating digital health literacy education, physical activity promotion, responsible social media use, and early identification of disordered eating.

## 1 Introduction

Disordered eating (DE) refers to a spectrum of maladaptive eating-related attitudes and behaviors, including excessive dietary restraint, preoccupation with food and weight, bulimic tendencies, and rigid control over eating. Adolescence is a particularly vulnerable period for the emergence of these symptoms (1, 2). A global systematic review and meta-analysis found that 22.36% of children and adolescents screened positive for DE using the SCOFF instrument (3). In China, a national study reported a weighted screen-positive proportion of 21.18%, which decreased to 11.33% when a clinical-significance criterion was applied (4). DE symptoms are associated with depression, anxiety, physical complications, and other adverse health outcomes (2, 5). Despite this substantial burden, research has focused mainly on prevalence estimates and individual risk correlates. Less attention has been paid to modifiable cognitive and behavioral pathways that could inform mental health promotion and school-based public health interventions.

Digital health literacy (DHL) is the capacity to access, understand, evaluate, and apply health information in digital environments (6). The Health Literacy Skills Framework conceptualizes health literacy as an upstream capability that shapes health outcomes through information application, behavioral mediators, and contextual influences, including media environments (7). Applied to adolescent DE, this framework suggests two relevant behavioral pathways. Stronger DHL may support health-oriented PA by helping adolescents translate reliable exercise information into action, consistent with observed associations between adolescent health literacy and PA (8). However, PA is not uniformly protective because compulsive or weight-control-motivated exercise may accompany eating pathology (9, 10). Stronger DHL may also support more regulated social media use, whereas PSMU may relate to DE symptoms through appearance comparison, ideal-body internalization, body dissatisfaction, and emotional distress (11). Evidence further links higher DHL with lower problematic mobile social media use and identifies PA as a contributing pathway (12). The compensatory internet use model suggests that health-oriented PA may reduce social media use for coping by strengthening offline engagement and emotion regulation (13). Thus, PA and PSMU may operate independently and sequentially in linking DHL with adolescent DE symptoms.

Previous studies have examined PA and PSMU separately in relation to body image and adolescent DE symptoms (14–16). Recent research has begun to integrate DHL, PA, and PSMU, but this work did not extend the pathway to DE symptoms (12). Thus, whether PA and PSMU operate as independent and sequential pathways linking DHL with adolescent DE remains unclear. Guided by the Health Literacy Skills Framework and the compensatory internet use model, this study examined the association between DHL and adolescent DE symptoms and tested independent and serial indirect associations through PA and PSMU. This integrated approach may inform school-based strategies combining DHL education, health-oriented PA, responsible social media use, and early recognition of DE symptoms.

## 2 Literature review

### 2.1 Relationship between DHL and DE

From a health promotion perspective, health literacy is not merely a reserve of health knowledge, but also a cognitive and behavioral resource that supports individuals in identifying health risks, making informed health judgments, and actively responding to health-related problems. It plays an important role in helping adolescents develop positive lifestyles (17, 18). Previous studies have shown that individuals with higher levels of health literacy generally possess stronger capacities for health information evaluation and decision-making, which may help them identify, understand, and respond to health-related problems more effectively (19). In the field of DE, higher health literacy may help adolescents develop more appropriate coping strategies and help-seeking attitudes, whereas insufficient health literacy is associated with higher levels of DE symptoms (20–22).

In digital health contexts, DHL further emphasizes individuals’ ability to access, understand, evaluate, and apply health information from electronic sources, with particular attention to their capacity to judge online information sources, content authenticity, and practical applicability (6, 23). Compared with general health literacy, DHL may reduce adolescents’ vulnerability to misinformation by strengthening their critical understanding of online information related to diet, weight management, and body image, as well as their capacity for health-related decision-making. In this way, DHL may be associated with DE symptoms (24, 25). However, existing research has paid limited attention to the relationship between DHL and DE, particularly the empirical testing of the “information evaluation–behavioral choice” mechanism. Therefore, further investigation is needed to clarify the pathways through which DHL may influence DE among adolescents.

### 2.2 The mediating role of PA

Based on the health promotion model, PA, as a positive health-promoting behavior, may reduce adolescents’ tendency toward DE by improving physical functioning and psychological adaptation (26, 27). At the level of physical functioning, the endorphin hypothesis suggests that PA can promote the release of endogenous opioid peptides such as β-endorphin, thereby producing analgesic, pleasurable, and stress-relieving effects. These effects may improve physiological arousal states and reduce negative emotional states, thereby potentially attenuating DE symptoms (28). At the psychological level, PA may also reduce DE through pathways such as emotion regulation and improved body image. Previous studies have indicated that emotion regulation is closely related to DE, whereas regular PA can reduce the risk of depression and alleviate negative emotions, thereby decreasing the likelihood of DE (29, 30). Other evidence suggests that participation in PA may help individuals focus more on bodily functionality rather than appearance-based evaluation, which may lower DE symptom levels to some extent (31). Thus, PA is not merely a form of physical exercise, but also a health resource that connects physiological regulation with psychological adaptation, providing protective support for reducing DE symptoms among adolescents (15, 27).

DHL may promote adolescents’ exercise-related cognition and PA participation by improving their ability to access, evaluate, and apply online health information (32, 33). More specifically, the key mechanism through which DHL promotes PA lies in its capacity to enhance adolescents’ judgment of health information and behavioral decision-making, enabling them to translate online health knowledge into actual exercise behavior and thereby increase PA participation. On the one hand, from the perspective of health management, higher DHL may help adolescents use digital tools for goal setting, activity monitoring, and feedback-based adjustment, thereby improving their self-management of PA (34). On the other hand, from the perspective of behavioral motivation, DHL may also strengthen adolescents’ sense of health responsibility and their recognition of the value of exercise, encouraging the translation of health intentions into actual exercise behavior (35). Evidence from systematic reviews and meta-analyses indicates that higher digital or electronic health literacy can facilitate the translation of health information into behavior and is closely associated with health-promoting behaviors such as PA (36). Taken together, the core mechanism by which DHL increases PA participation lies in the transformation process of “health information acquisition–exercise value recognition–behavioral decision-making–self-management.” Through this process, adolescents may internalize online health knowledge into exercise motivation and implement it as sustained PA behavior. Therefore, PA may mediate the relationship between DHL and DE.

### 2.3 The mediating role of PSMU

PSMU refers to an uncontrolled pattern of social media use characterized by excessive preoccupation with social media, strong urges to use it, and difficulty controlling use, even when such behavior has already impaired daily learning, sleep, or social functioning (37). Existing evidence suggests that the relationship between PSMU and DE is not merely linear, but may involve the cumulative effects of multiple pathways, such as disrupted sleep rhythms and biased appearance-related cognition (38, 39). Specifically, at the lifestyle level, PSMU is often accompanied by difficulties falling asleep, reduced sleep quality, and irregular daily routines. Insufficient sleep may further disrupt biological rhythms, thereby being associated with higher levels of DE symptoms (40). At the psychological and cognitive level, PSMU may increase adolescents’ exposure to appearance-, body shape-, and weight-control-related content, and may reinforce body dissatisfaction through upward social comparison, thereby exacerbating DE-related symptoms (16). In addition, PSMU is often accompanied by negative emotions and emotion dysregulation, and difficulties in emotion regulation are themselves important factors associated with DE (30, 41). Thus, PSMU may be associated with higher levels of DE symptoms through both physiological and psychological mechanisms.

As a key capacity for adolescents to navigate complex digital media environments, DHL may reduce the tendency toward PSMU by enhancing adolescents’ ability to evaluate the credibility of online health information, identify risks, and regulate their own media use, thereby interrupting the transition from information seeking to dependent use (42, 43). A systematic review and meta-analysis showed that PSMU is significantly associated with adverse mental health outcomes, including stress, depression, anxiety, and sleep problems, indicating that it has become an important issue in research on adolescent digital media use risks (37). Another study suggested that higher levels of DHL may help adolescents more effectively identify and cope with psychological risks in information-overloaded digital environments, thereby reducing tendencies toward social media addiction (44). Accordingly, PSMU may mediate the relationship between DHL and DE, constituting an important psychological and behavioral pathway for reducing DE symptoms among adolescents.

### 2.4 The serial effect of PA and PSMU

Based on the compensatory internet use theory, PA may reduce adolescents’ need for compensatory social media use by improving emotion regulation and real-life adaptation, thereby lowering the risk of PSMU (13). Consistent with this mechanism, a cross-sectional questionnaire study in China found that adolescents with higher levels of PA in the past seven days reported lower levels of social networking site addiction, and that this association operated through a serial pathway involving anxiety and ego depletion. This suggests that PA may weaken adolescents’ dependence on social media by alleviating emotional distress and improving self-regulation (45). Empirical studies have also shown that PA may buffer against PSMU by reducing sedentary screen behavior, promoting offline social participation, and improving emotion regulation, whereas adolescents with insufficient PA may face a higher risk of PSMU (46). Therefore, PA may reduce the risk of PSMU through pathways involving emotion regulation, real-life adaptation, and self-control, which may explain the association between higher PA levels and lower tendencies toward PSMU.

From the perspective of behavioral and psychological risk accumulation and its eventual influence on health outcomes, PA and PSMU may serve as two key mediating variables in the association between DHL and adolescent DE (12). Specifically, from the perspective of health behavior promotion, adolescents with higher DHL generally show more favorable levels of health promotion. They tend to perform better in domains such as exercise and health responsibility, and are more likely to translate online health information into stable PA and self-management behaviors (35, 47). Furthermore, PA may reduce adolescents’ dependence on social media by alleviating anxiety, reducing ego depletion, and enhancing self-regulation. A lower level of PSMU may then reduce exposure to appearance comparison, body dissatisfaction, and diet-control content, which may, in turn, be associated with lower levels of DE symptoms (16, 45). Although existing studies have not directly tested the complete serial pathway linking DHL, PA, PSMU, and DE, they provide important theoretical support for the present study. Accordingly, PA and PSMU may play a serial mediating role in the relationship between DHL and adolescent DE.

### 2.5 Research hypotheses

**Figure 1:**
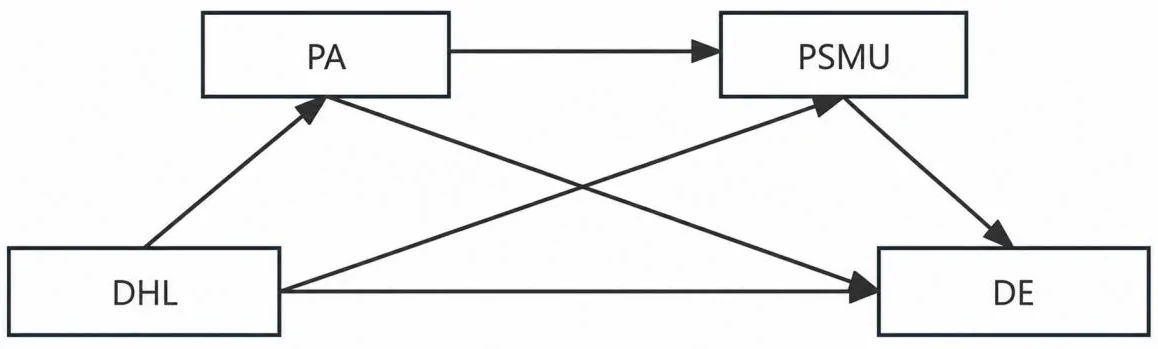
Hypothesized serial mediation model of physical activity and problematic social media use in the association between digital health literacy and disordered eating. DHL, Digital health literacy; DE, Disordered Eating; PA, Physical activity; PSMU, Problematic social media use.

This study aimed to examine the mediating roles of PA and PSMU in the relationship between DHL and DE among adolescents, thereby deepening understanding of how DHL may be associated with adolescent DE through health behaviors and media-use behaviors. Previous studies have shown that DHL, PA, and PSMU are closely related to adolescents’ eating attitudes, body image, and DE-related problems. However, empirical evidence on how these factors jointly relate to adolescent DE remains relatively limited. Therefore, this study sought to clarify the relationship between DHL and adolescent DE, with particular attention to the potential independent mediating pathways and serial mediating pathway involving PA and PSMU. The findings are expected to provide a theoretical basis for risk identification and the promotion of adolescents’ physical and mental health development in relation to DE. Based on the existing literature, the following four hypotheses were proposed (see **Figure 1**):

H1: DHL is negatively associated with adolescent DE.
H2: PA independently mediates the relationship between DHL and adolescent DE.
H3: PSMU independently mediates the relationship between DHL and adolescent DE.
H4: PA and PSMU play a serial mediating role in the relationship between DHL and adolescent DE.

## 3 Methods

### 3.1 Participants

**Figure 2:**
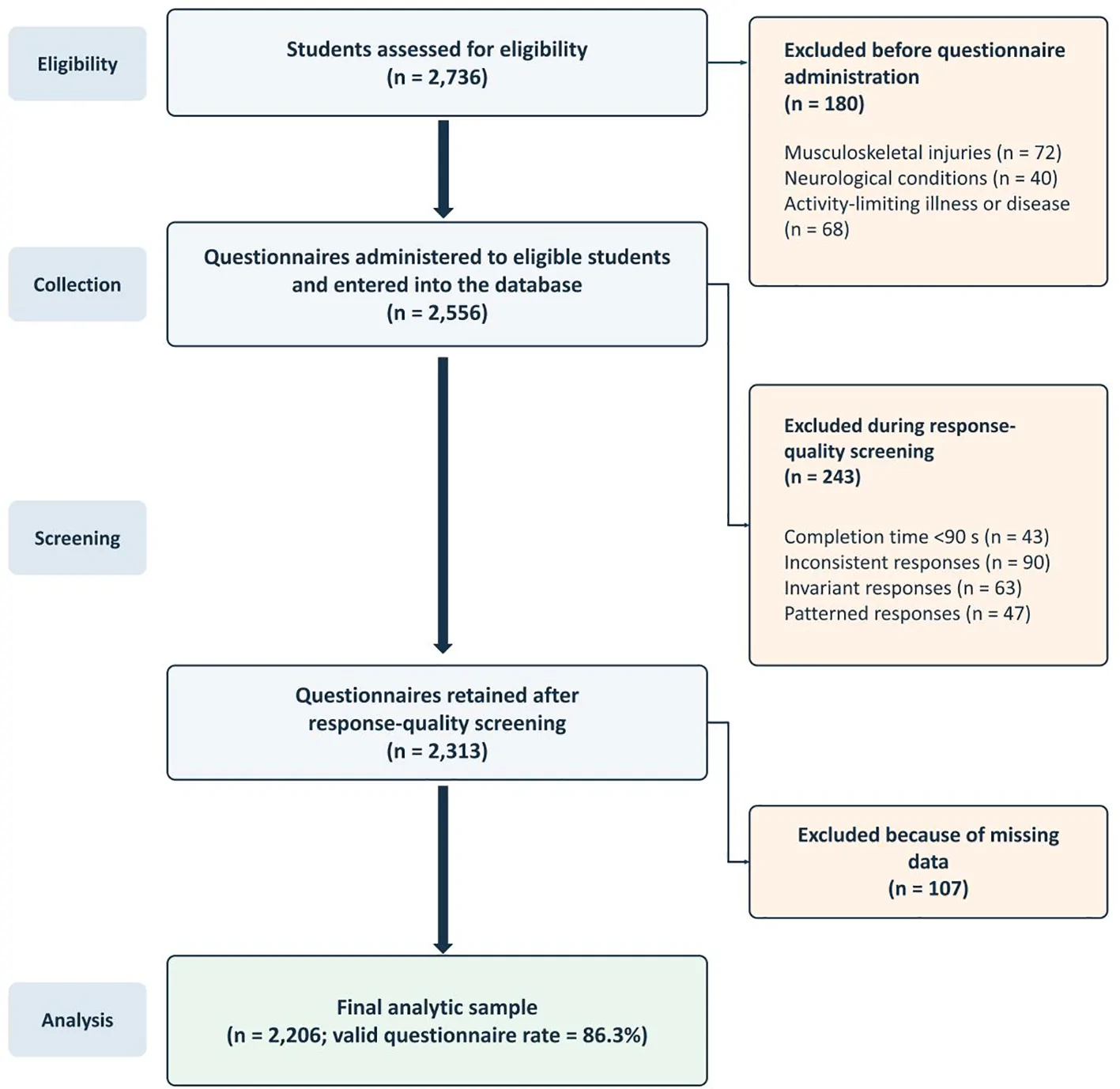
Steps in the screening process for research samples.

This cross-sectional study recruited participants from five secondary schools located in urban areas of Henan Province, China, including three public schools and two private schools. Detailed characteristics of the participating schools are presented in **Supplementary Table 1**. Before questionnaire administration, 2,736 students were screened for eligibility based on self-reported health status; 180 reporting activity-limiting health conditions were excluded, leaving 2,556 eligible students. Questionnaires were distributed to all 2,556 eligible students, of which 2,206 valid responses were retained for the final analysis, yielding a valid response rate of 86.3%. The detailed screening procedure is shown in **Figure 2**. To evaluate the adequacy of the achieved sample size, a Monte Carlo sensitivity analysis was conducted for the hypothesized serial mediation model. With *N* = 2,206 and *α* = 0.05, the study had 95% power to detect a standardized serial indirect effect as small as 0.0008, supporting the adequacy of the sample size under the specified model assumptions (48). Among the 2,206 valid respondents, 1,100 were boys (49.9%) and 1,106 were girls (50.1%). The mean age of the participants was 14.50 ± 1.76 years. Detailed demographic characteristics are presented in **Table 1**.

**Table 1:** Distribution of basic information on adolescents (*N* = 2206).

Demographic variables		Number	Proportion%
Age	14.50 ± 1.76	2206	100%
Sex	Male	1100	49.9%
	Female	1106	50.1%
Grade level	Middle school	1057	47.9%
	High school	1149	52.1%
Place of birth	Rural	933	42.3%
	Urban	1273	57.7%
Only child (yes/no)	Yes	491	22.3%
	No	1715	77.7%
Boarding student (yes/no)	Yes	1509	68.4%
	No	697	31.6%

To ensure that the sample was aligned with the aims of the study and to maintain data quality, clear inclusion and exclusion criteria were established in advance according to the research objectives and measurement requirements. The inclusion criteria were as follows: (1) full-time junior or senior secondary school students currently enrolled in school; (2) normal cognitive functioning and reading comprehension, enabling participants to complete the self-report questionnaires independently and accurately; and (3) voluntary participation, with written informed consent obtained from the participants’ parents or legal guardians and written assent obtained from the adolescents themselves. The exclusion criteria were as follows: (1) recent physical trauma or medically advised contraindications to exercise; (2) a confirmed diagnosis of other neurodevelopmental disorders or neurological diseases; and (3) acute or chronic diseases.

### 3.2 Research procedure

This study was conducted in Henan Province, China, from March to April 2026. Purposive sampling was used at the school level, whereas convenience sampling was used to recruit eligible students within the participating schools. The study was approved by the Biomedical Research Ethics Committee of Henan University (Approval No. HUSOM2026-617). Before data collection, permission was obtained from the local education authorities and participating schools. Written informed consent was obtained from the parents or legal guardians of all minor participants, and the adolescents themselves voluntarily provided written assent after receiving age-appropriate information about the study.

The questionnaires were administered in classroom settings by trained research assistants, who provided standardized instructions and answered procedural questions. Before completing the questionnaires, participants were informed of the study purpose, the voluntary nature of participation, the anonymity of their responses, the confidentiality of the collected data, and their right to withdraw. Participants completed the questionnaires independently and returned them immediately after completion. Each questionnaire was checked for completeness upon submission, and any unintentionally omitted items were confirmed with the participant before final collection.

### 3.3 Measures

#### 3.3.1 Digital health literacy

DHL was assessed using the simplified Chinese version of the eight-item eHealth Literacy Scale (eHEALS), translated and culturally adapted by (49).For Chinese senior high school students. The eHEALS consists of eight items covering three content domains: applying online health information and services, evaluating online health information, and making health-related decisions. Items are rated on a five-point Likert scale ranging from 1 (“strongly disagree”) to 5 (“strongly agree”). The total score is obtained by summing the item scores and ranges from 8 to 40, with higher scores indicating higher levels of self-reported DHL. Previous research has demonstrated good reliability and validity of this version among Chinese adolescents (50, 51). In the present study, Cronbach’s alpha was 0.83, indicating good internal consistency.

#### 3.3.2 Disordered eating

DE was assessed using the Eating Attitudes Test-26 (EAT-26), developed by (52). The EAT-26 is rated on a six-point Likert scale and includes 26 items across three dimensions: dieting, bulimia and food preoccupation, and oral control. All items were scored in the same direction, and the total score was obtained by summing the item scores. Higher scores indicate a greater likelihood of abnormal eating attitudes and behaviors. Previous studies have demonstrated good internal consistency of the EAT-26 in Chinese populations (53). In the present study, Cronbach’s alpha was 0.92, indicating good internal consistency.

#### 3.3.3 Physical activity

PA was assessed using the Physical Activity Rating Scale-3 (PARS-3), revised by (54). The PARS-3 comprises three items assessing exercise intensity, duration, and frequency over the previous month. Intensity and frequency are scored from 1 to 5, whereas duration is recoded from 0 to 4. The total PA score is calculated as intensity × recoded duration × frequency, yielding a range of 0–100. Scores of ≤19, 20–42, and ≥43 indicate low, moderate, and high PA levels, respectively. Previous studies in Chinese adolescent samples have shown that the PARS-3 has good internal consistency among secondary school students (55, 56). In the present study, Cronbach’s alpha was 0.79, indicating that the scale was suitable for assessing adolescents’ physical exercise levels.

#### 3.3.4 Problematic social media use

PSMU was assessed using the Bergen Social Media Addiction Scale (BSMAS) developed by (57). The BSMAS is rated on a five-point Likert scale, ranging from 1 (“never”) to 5 (“always”). The scale consists of six items. The total score is calculated by summing the item scores, with possible scores ranging from 6 to 30. Higher scores indicate stronger tendencies toward social media addiction. The BSMAS has been widely used in Chinese populations and has demonstrated good internal consistency (58). In the present study, Cronbach’s alpha was 0.94, indicating good internal consistency.

### 3.4 Data analysis

Statistical analyses were performed using SPSS 27.0. The significance level for two-tailed tests was set at p < 0.05. First, the valid data were organized and checked for quality, and Harman’s single-factor test was used to assess the potential influence of common method bias. Descriptive statistics were then conducted for DHL, PA, PSMU, and DE, with means and standard deviations used to describe the distribution of each variable. Pearson correlation analysis was used to examine the correlations among the main variables. To further examine the predictive relationships among variables, hierarchical multiple linear regression models were constructed, and multicollinearity diagnostics were conducted before regression analysis. Finally, Model 6 of the PROCESS macro developed by Hayes was used to test the serial mediation effect. Mediation effects were examined using the Bootstrap method with 5,000 resamples, and 95% confidence intervals were calculated. An indirect effect was considered statistically significant if the confidence interval did not include zero.

## 4 Results

### 4.1 Common method bias test

Because all variables were assessed using self-report scales, Harman’s single-factor test was conducted to evaluate the potential influence of common method bias. The results showed that 13 common factors with eigenvalues greater than 1 were extracted through principal component analysis, collectively explaining 87.36% of the total variance. The first unrotated factor accounted for 27.18% of the variance, which was well below the threshold of 40%. This indicates that there was no serious common method bias in the data.

### 4.2 Descriptive statistics and correlation analysis

**Table 2** presents the means, standard deviations, and correlations among DHL, DE, PA, and PSMU. The mean (SD) scores were 31.38 (6.86) for DHL, 7.88 (7.09) for DE, 27.02 (23.96) for PA, and 15.07 (7.94) for PSMU. Correlation analyses showed that DHL was negatively correlated with DE (*r* = −0.314, *p* < 0.01) and PSMU (*r* = −0.378, *p* < 0.01) and positively correlated with PA (*r* = 0.365, *p* < 0.01). PA was negatively correlated with DE (*r* = −0.384, *p* < 0.01) and PSMU (*r* = −0.328, *p* < 0.01), whereas PSMU was positively correlated with DE (*r* = 0.369, *p* < 0.01).

**Table 2:** Means, standard deviations and correlations among all variables (*N* = 2206).

Variable	*M*	*SD*	1	2	3	4
1. Digital health literacy	31.38	6.86	**1**	-0.314^**^	0.365^**^	-0.378^**^
2. Disordered eating	7.88	7.09	-0.314^**^	**1**	-0.384^**^	0.369^**^
3. Physical activity	27.02	23.96	0.365^**^	-0.384^**^	**1**	-0.328^**^
4. Problematic social media use	15.07	7.94	-0.378^**^	0.369^**^	-0.328^**^	**1**

**p < 0.01 (two-tailed). Bold values indicate self-correlations on the diagonal (r = 1.00).

### 4.3 Regression analysis

To examine whether DHL, PA, and PSMU could predict DE, hierarchical multiple regression analysis was conducted. The results are presented in **Table 3**. Before the regression analysis, multicollinearity diagnostics were performed for all predictor variables. The results showed that the variance inflation factor (VIF) values ranged from 1.565 to 2.073, which were far below the threshold of 10. This indicates that there was no serious multicollinearity in the data and that the variables were suitable for subsequent regression and mediation analyses.

**Table 3:** Standardized regression coefficients and model-fit statistics for the regression models.

Variable		Dependent variable: DE								Dependent variable: PA		Dependent variable: PSMU	
		Model 1		Model 2		Model 3		Model 4		Model 5		Model 6	
		β	*t*	β	*t*	β	*t*	β	*t*	β	*t*	β	*t*
Control variables	Sex	0.02	0.82	0.01	0.56	-0.02	-0.86	-0.01	-0.36	-0.09	-4.54***	-0.04	-2.17*
	Age	-0.05	-2.50**	-0.06	-2.76**	-0.04	-2.18**	-0.05	-2.65**	0.05	2.24*	0.03	1.70
	Grade level	-0.04	-1.92	-0.03	-1.34	-0.01	-0.60	-0.02	-0.94	0.05	2.51	0.03	1.30
	Place of birth	-0.01	-0.09	0.01	0.41	0.01	0.70	0.01	0.69	0.02	0.82	0.01	0.11
	Only child or not	-0.03	-1.52	-0.01	-0.51	0.02	1.07	0.01	0.08	0.11	5.01***	0.09	4.22**
	Boarding or not	0.02	-0.89	-0.03	-1.51	-0.03	-1.60	-0.04	-2.09	0.00	-0.04	0.04	1.83
Independent variable	DHL	–	–	-0.32	-15.50***	-0.20	-9.84***	-0.13	-6.32***	0.35	17.86***	-0.30	-14.50***
Mediating variables	PA	–	–	–	–	-0.32	-15.08***	-0.26	-12.43***	–	–	-0.23	-11.25***
	PSMU	–	–	–	–	–	–	0.24	11.35***	–	–	–	–
Model fit	*R*	0.08		0.32		0.43		0.48		0.39		0.44	
	*R^2^*	0.01		0.10		0.19		0.23		0.15		0.19	
	Adjusted R^2^	0.01		0.10		0.19		0.23		0.15		0.19	
	*F*	2.18*		36.38***		63.52***		74.06***		57.59***		66.11***	

DHL, Digital health literacy; DE, Disordered eating; PA, Physical activity; PSMU, Problematic social media use. *p < 0.05, **p < 0.01, ***p < 0.001. Complete regression results, including unstandardized coefficients (B), standard errors (SE), 95% confidence intervals for B, and exact p values, are presented in **Supplementary Table 2**.

First, in Model 1, demographic variables, including sex, age, grade, place of birth, and only-child status, were entered as control variables. Subsequently, DHL was added in Model 2. The results showed that DHL significantly predicted lower levels of DE (β = -0.32, *p* < 0.001), supporting H1. In addition, the results of Model 3 indicated that PA significantly predicted lower levels of DE (β = -0.32, *p* < 0.001). In Model 4, PSMU was further found to significantly predict higher levels of DE (β = 0.24, *p* < 0.001).

Furthermore, as shown in Model 5, DHL had a significant positive association with PA (β = 0.35, p < 0.001). Finally, the results of Model 6 showed that PA significantly negatively predicted PSMU (β = -0.23, *p* < 0.001). Overall, the hierarchical regression analysis clarified the associations among DHL, PA, and PSMU, and all key variables showed significant predictive associations with DE.

### 4.4 Mediation analysis

**Figure 3:**
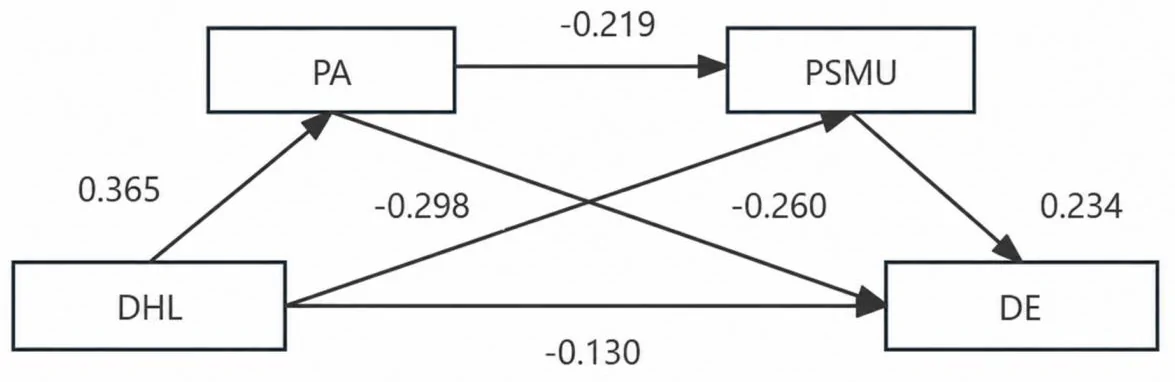
Serial mediation model of physical activity and problematic social media use in the association between digital health literacy and disordered eating, with standardized path coefficients. DHL, Digital health literacy; DE, Disordered eating; PA, Physical activity; PSMU, Problematic social media use.

The standardized path coefficients of the serial mediation model are shown in **Figure 3**, and the results of the mediation analysis are presented in **Table 4**. The estimated direct effect of DHL on adolescent DE was statistically significant (*b* = -0.135, bootstrap 95% CI [-0.177, -0.092]), accounting for 41.7% of the total effect. The total indirect effect through PA and PSMU was also statistically significant (*b* = -0.190, bootstrap 95% CI [-0.218, -0.163]), accounting for 58.6% of the total effect. Specifically, the serial indirect effect through PA and PSMU was -0.019 (bootstrap 95% CI [-0.024, -0.015]), accounting for 5.9% of the total effect and supporting the proposed serial mediation pathway.

**Table 4:** Mediating effects and effect sizes.

Path	Effect	*SE*	Bootstrap 95% CI		Percentage of total effect
			Lower	Upper	
Total effect	-0.324	0.021	-0.365	-0.283	–
Direct Effect	-0.135	0.022	-0.177	-0.092	41.7%
Total indirect effects	-0.190	0.014	-0.218	-0.163	58.6%
Ind_1_: DHL→PA→DE	-0.098	0.011	-0.121	-0.077	30.2%
Ind_2_: DHL→PSMU→DE	-0.072	0.008	-0.089	-0.057	22.2%
Ind_3_: DHL→PA→PSMU→DE	-0.019	0.002	-0.024	-0.015	5.9%

Specifically, the mediation analysis for Ind1 showed that PA significantly mediated the relationship between DHL and adolescent DE, with an indirect effect of -0.098 and a 95% CI of [-0.121, -0.077], accounting for 30.2% of the total effect. This result supported H2. Further analysis showed that PSMU also significantly mediated the relationship between DHL and adolescent DE, with an indirect effect of -0.072 and a 95% CI of [-0.089, -0.057], accounting for 22.2% of the total effect. This result supported H3. In addition, the serial mediating effect of PA and PSMU in the relationship between DHL and adolescent DE was also significant, with an indirect effect of -0.019 and a 95% CI of [-0.024, -0.015], accounting for 5.9% of the total effect. This result supported H4.

## 5 Discussion

As health information seeking and social interaction have become increasingly digitalized, the online environment in which adolescents are embedded not only provides a new context for shaping health behaviors, but may also increase vulnerability to DE through PSMU. Against this background, the present study examined the relationships among DHL, PA, PSMU, and DE. The findings showed that higher DHL was significantly associated with lower levels of DE symptoms. This association may not be limited to information identification alone, but may also operate indirectly through lower levels of PSMU and greater engagement in health-promoting behaviors such as PA.

### 5.1 The association between DHL and DE

The results of this study showed that DHL was significantly negatively associated with DE among adolescents, indicating that adolescents with higher DHL tended to report fewer DE symptoms. This finding supports H1. Consistent with this result, Boberová and Husárová (20) found that lower health literacy was associated with higher levels of DE symptoms among adolescents, providing supporting evidence from another cultural context (20). The present findings are therefore consistent with a potentially protective role of DHL in digital health environments. One possible explanation is that DHL may enhance adolescents’ ability to evaluate the credibility of information about diet, weight loss, and body image and to critically identify misleading content. This may limit the internalization of narrow body ideals and extreme weight-control beliefs, thereby potentially attenuating DE symptoms (24, 59). At the same time, individual- and family-level factors, including sex, age, body image, and family socioeconomic background, may be associated with both adolescents’ DHL and DE symptoms. The association remained significant after adjustment for the measured covariates, suggesting that it was not fully accounted for by these factors, although residual confounding cannot be excluded (60, 61). Given adolescents’ extensive engagement with social media, DHL may help them maintain a critical stance toward media content shaped by algorithmic recommendations, commercial communication, and peer evaluation. This may reduce their identification with and internalization of idealized body standards, thereby potentially buffering against DE symptoms (62). These findings suggest that schools and families may consider integrating DHL development into routine health education and psychological support, helping adolescents critically evaluate inaccurate weight-loss information, develop more evidence-based dietary knowledge, and maintain more positive body attitudes. Nevertheless, because of the cross-sectional design, the direction and causality of the observed association cannot be established.

### 5.2 The mediating role of PA between DHL and DE

The mediation analysis indicated that PA partially accounted for the association between DHL and DE, supporting H2. Pender’s health promotion model proposes that health behaviors are shaped by knowledge, perceived benefits, self-efficacy, and contextual support. From this perspective, DHL may help adolescents recognize the benefits of PA, evaluate opportunities for participation, and make informed health decisions (63, 64). Evidence from adult populations similarly links the ability to understand, evaluate, and apply digital health information with greater PA participation (65). Among adolescents, however, PA participation may also depend on school physical education, family support, and peer interaction. Consistent with this interpretation, insufficient health literacy among adolescents has been associated with lower MVPA (8). Together, this evidence provides a plausible basis for the observed indirect association between DHL and DE through PA.

The negative association between PA and DE symptoms may reflect physiological and psychological processes related to energy regulation and body functionality (66, 67). Physiologically, regular PA during adolescence may support energy balance by influencing appetite- and metabolism-related hormones, including leptin and adiponectin, while improving body composition and perceived body functionality (15, 68). Psychologically, enhanced body functionality, exercise self-efficacy, and positive affect may reduce weight preoccupation, body dissatisfaction, and appearance anxiety (56, 69). These mechanisms remain interpretive because they were not directly assessed in the present study. Moreover, PA should not be regarded as uniformly protective, as excessive, compulsive, or primarily weight-control-motivated exercise may accompany DE symptoms (9, 10). Because the PARS-3 measures overall exercise volume without assessing motivation or compulsivity, the findings cannot exclude harmful exercise patterns or nonlinear associations. Interventions should therefore promote health-oriented and appropriately regulated PA rather than simply encouraging greater exercise volume.

### 5.3 The mediating role of PSMU between DHL and DE

The mediation analysis identified a significant indirect association between DHL and DE through PSMU, supporting H3. From an executive-control perspective, DHL may help adolescents recognize social media-related risks and regulate impulsive or uncontrolled use, thereby limiting PSMU (70, 71). This interpretation is consistent with the inverse association observed in the present study and the potentially protective role of DHL reported previously (12). DHL may also help adolescents identify emotion-driven use and dependence on online feedback, enabling more informed decisions about social media engagement (72, 73). Accordingly, stronger DHL may be associated with lower PSMU through improved risk recognition, information evaluation, and behavioral self-regulation.

PSMU was positively associated with DE symptoms, suggesting that it may represent a risk-related factor among adolescents. Consistent with this finding, Toğuç (74) reported weak positive correlations between PSMU and binge eating and purging among Turkish university students (74). Although that population differed from the present sample, the findings provide complementary evidence linking PSMU to specific DE behaviors. From an emotion-regulation perspective, PSMU may involve greater dependence on online recognition and feedback, together with heightened negative emotional arousal. Popularity- and appearance-related motives for social media use have also been associated with greater DE symptoms (75). Negative affect related to problematic use may encourage restrictive, emotional, or binge eating as coping responses (76–78). Together, these processes provide a plausible explanation for the observed indirect association between DHL and DE through PSMU. However, the temporal and causal ordering of these associations cannot be established from the present cross-sectional data.

### 5.4 The serial mediating effect of PA and PSMU between DHL and DE

The analysis showed that DHL was indirectly associated with DE through the serial indirect pathway involving PA and PSMU, supporting H4. From an emotion-regulation perspective, PA may support emotion regulation, stress relief, and real-life engagement. PA was negatively associated with PSMU among adolescents, suggesting that adolescents with higher PA levels generally reported lower tendencies toward PSMU (45). From the perspective of behavioral choice, adolescents with higher PA levels may be less likely to use digital media as an alternative emotion-regulation strategy or a source of immediate gratification (79). From the perspective of emotional coping, these adolescents may be more inclined to relieve stress and restore emotional balance through PA rather than social media. Consequently, they may show less reliance on social media for emotional compensation and lower levels of PSMU (80–82). Thus, PA may be associated with lower emotional reliance on digital media by providing an adaptive emotion-regulation pathway, potentially contributing to lower PSMU.

As a key enabling resource in the digital era, DHL may be indirectly associated with fewer DE symptoms through a proposed serial pathway involving greater PA and lower PSMU. However, because the present study was cross-sectional, this pathway should be interpreted as a theory-consistent statistical pattern rather than a confirmed temporal or causal mechanism. The findings suggest that the prevention and early identification of adolescent DE may benefit from moving beyond single psychological or diet-focused approaches. A more integrated approach could combine DHL development, health-oriented PA promotion, and guidance on healthy social media use. Schools and public health systems may therefore consider integrating digital health education into physical education curricula and mental health services. Such integration may support adolescents’ critical evaluation of health information, offline PA participation, and more regulated social media use. These efforts may provide a practical basis for the early identification and comprehensive prevention of DE symptoms.

### 5.5 Limitations and future directions

Several limitations should be acknowledged. First, the cross-sectional design precludes causal inference, while the reliance on self-reported measures may have introduced recall and social-desirability biases. Second, although the PARS-3 provides an estimate of overall exercise volume, it does not capture detailed activity types or distinguish compulsory physical education from extracurricular activity. We also did not assess the duration or content of social media use, preventing dose- or content-specific analyses. Third, students were nested within five schools, but school-level clustering was not modelled. Unaccounted within-school correlations may therefore have resulted in underestimated standard errors. Fourth, the purposive and convenience sampling procedures, recruitment from urban secondary schools in a single province, and exclusion of students with activity-limiting health conditions may have introduced selection bias and limited the generalizability of the findings. Future studies should use longitudinal designs, recruit larger numbers of schools through probability-based sampling, apply multilevel analyses, and incorporate more detailed or objective measures of physical activity and social media use.

Future studies should use longitudinal or intervention designs to clarify the temporal and potentially causal pathways linking DHL to DE symptoms. Measurement could be strengthened by combining self-reports with objective activity monitoring, smartphone logs, and parent or teacher reports. Research should also distinguish social media exposure by duration and content, particularly body image, dieting, weight-loss, and algorithmically recommended material. More detailed assessments of PA type, participation motives, and school-level physical education support may further clarify how different activity contexts relate to DHL and DE.

## 6 Conclusion

This study constructed a serial mediation model with PA and PSMU as mediators to examine the pathways linking DHL and adolescent DE. The results showed that DHL was significantly negatively associated with DE, providing new insights into the pathway associated with related to adolescent DE in digital contexts. PA and PSMU played both independent mediating roles and a serial mediating role in the relationship between DHL and DE. Specifically, higher levels of DHL were associated with greater PA participation, which in turn was associated with lower levels of PSMU, thereby being indirectly associated with lower levels of DE symptoms. These findings highlight the key roles of DHL, PA, and PSMU in the prevention of adolescent DE and provide empirical evidence for school health education, PA promotion, and digital media interventions.

## Data Availability

The raw data supporting the conclusions of this article will be made available by the authors, without undue reservation.
