# Supplementary material for: Digital health literacy and disordered eating among adolescents: the chain mediating roles of physical activity and problematic social media use
Source: Front Psychiatry. 2026 Aug 19;17:1937781. doi: 10.3389/fpsyt.2026.1937781 (PMC13533913; doi:10.3389/fpsyt.2026.1937781)
Supplement: Supplementary file 1 [file Table1.docx]

**Supplementary Table S1. Distribution of participants across participating schools (N = 2,206)**

| **School** | **School type** | **Total, n (%)** | **Middle school, n (%)** | **High school, n (%)** | **Age, years, mean ± SD** |
| --- | --- | --- | --- | --- | --- |
| School A | Public | 492 (22.3) | 238 (48.4) | 254 (51.6) | 14.45 ± 1.75 |
| School B | Public | 463 (21.0) | 221 (47.7) | 242 (52.3) | 14.52 ± 1.77 |
| School C | Public | 441 (20.0) | 209 (47.4) | 232 (52.6) | 14.48 ± 1.74 |
| School D | Private | 418 (18.9) | 201 (48.1) | 217 (51.9) | 14.56 ± 1.78 |
| School E | Private | 392 (17.8) | 188 (48.0) | 204 (52.0) | 14.50 ± 1.76 |
| Total | 3 public; 2 private | 2,206 (100.0) | 1,057 (47.9) | 1,149 (52.1) | 14.50 ± 1.76 |
