# Supplementary material for: Digital health literacy and disordered eating among adolescents: the chain mediating roles of physical activity and problematic social media use
Source: Front Psychiatry. 2026 Aug 19;17:1937781. doi: 10.3389/fpsyt.2026.1937781 (PMC13533913; doi:10.3389/fpsyt.2026.1937781)
Supplement: Supplementary file 2 [file Table2.docx]

**Supplementary Table S2. Complete regression coefficients for the six regression models.**

**Panel A. Hierarchical models with DE as the dependent variable**

| **Model** | **Predictor** | **B** | **SE** | **β** | **95% CI for B** | **t** | **p** |
| --- | --- | --- | --- | --- | --- | --- | --- |
| Model 1 | Constant | 12.109 | 1.446 | - | [9.273, 14.944] | 8.374 | <0.001 |
|  | Sex | 0.251 | 0.305 | 0.018 | [-0.348, 0.849] | 0.821 | 0.412 |
|  | Age | -0.177 | 0.071 | -0.054 | [-0.316, -0.038] | -2.502 | 0.012 |
|  | Grade level | -0.597 | 0.311 | -0.042 | [-1.207, 0.013] | -1.918 | 0.055 |
|  | Place of birth | -0.027 | 0.312 | -0.002 | [-0.639, 0.585] | -0.085 | 0.932 |
|  | Only-child status | -0.588 | 0.387 | -0.034 | [-1.346, 0.171] | -1.520 | 0.129 |
|  | Boarding status | -0.298 | 0.336 | -0.020 | [-0.957, 0.362] | -0.885 | 0.376 |
| Model 2 | Constant | 21.547 | 1.502 | - | [18.601, 24.493] | 14.343 | <0.001 |
|  | Sex | 0.164 | 0.290 | 0.012 | [-0.405, 0.732] | 0.564 | 0.573 |
|  | Age | -0.186 | 0.067 | -0.057 | [-0.318, -0.054] | -2.764 | 0.006 |
|  | Grade level | -0.397 | 0.296 | -0.028 | [-0.976, 0.183] | -1.341 | 0.180 |
|  | Place of birth | 0.122 | 0.296 | 0.008 | [-0.459, 0.703] | 0.411 | 0.681 |
|  | Only-child status | -0.188 | 0.368 | -0.011 | [-0.910, 0.534] | -0.511 | 0.610 |
|  | Boarding status | -0.483 | 0.320 | -0.032 | [-1.110, 0.144] | -1.511 | 0.131 |
|  | DHL | -0.325 | 0.021 | -0.315 | [-0.366, -0.284] | -15.497 | <0.001 |

**Panel A (continued). Hierarchical models with DE as the dependent variable**

| **Model** | **Predictor** | **B** | **SE** | **β** | **95% CI for B** | **t** | **p** |
| --- | --- | --- | --- | --- | --- | --- | --- |
| Model 3 | Constant | 19.037 | 1.440 | - | [16.213, 21.861] | 13.220 | <0.001 |
|  | Sex | -0.240 | 0.277 | -0.017 | [-0.784, 0.304] | -0.864 | 0.387 |
|  | Age | -0.140 | 0.064 | -0.043 | [-0.266, -0.014] | -2.181 | 0.029 |
|  | Grade level | -0.170 | 0.282 | -0.012 | [-0.723, 0.383] | -0.602 | 0.547 |
|  | Place of birth | 0.197 | 0.282 | 0.014 | [-0.357, 0.750] | 0.696 | 0.487 |
|  | Only-child status | 0.376 | 0.352 | 0.022 | [-0.315, 1.068] | 1.068 | 0.286 |
|  | Boarding status | -0.487 | 0.304 | -0.032 | [-1.084, 0.109] | -1.601 | 0.109 |
|  | DHL | -0.210 | 0.021 | -0.204 | [-0.252, -0.169] | -9.844 | <0.001 |
|  | PA | -0.093 | 0.006 | -0.315 | [-0.105, -0.081] | -15.075 | <0.001 |
| Model 4 | Constant | 14.170 | 1.464 | - | [11.299, 17.041] | 9.679 | <0.001 |
|  | Sex | -0.098 | 0.270 | -0.007 | [-0.627, 0.431] | -0.363 | 0.717 |
|  | Age | -0.166 | 0.062 | -0.050 | [-0.288, -0.043] | -2.652 | 0.008 |
|  | Grade level | -0.256 | 0.274 | -0.018 | [-0.794, 0.281] | -0.935 | 0.350 |
|  | Place of birth | 0.190 | 0.274 | 0.013 | [-0.349, 0.728] | 0.691 | 0.490 |
|  | Only-child status | 0.026 | 0.344 | 0.002 | [-0.649, 0.701] | 0.076 | 0.939 |
|  | Boarding status | -0.618 | 0.296 | -0.041 | [-1.199, -0.038] | -2.088 | 0.037 |
|  | DHL | -0.138 | 0.022 | -0.133 | [-0.180, -0.095] | -6.320 | <0.001 |
|  | PA | -0.077 | 0.006 | -0.260 | [-0.089, -0.065] | -12.432 | <0.001 |
|  | PSMU | 0.211 | 0.019 | 0.236 | [0.175, 0.247] | 11.351 | <0.001 |

**Panel B.Regression models predicting PA and PSMU**

| **Model** | **Predictor** | **B** | **SE** | **β** | **95% CI for B** | **t** | **p** |
| --- | --- | --- | --- | --- | --- | --- | --- |
| Model 5 | Constant | -26.899 | 4.929 | - | [-36.566, -17.232] | -5.457 | <0.001 |
|  | Sex | -4.322 | 0.951 | -0.090 | [-6.188, -2.457] | -4.543 | <0.001 |
|  | Age | 0.494 | 0.221 | 0.045 | [0.061, 0.927] | 2.237 | 0.025 |
|  | Grade level | 2.432 | 0.970 | 0.051 | [0.529, 4.334] | 2.506 | 0.012 |
|  | Place of birth | 0.799 | 0.973 | 0.016 | [-1.109, 2.707] | 0.821 | 0.412 |
|  | Only-child status | 6.048 | 1.208 | 0.105 | [3.679, 8.416] | 5.007 | <0.001 |
|  | Boarding status | -0.047 | 1.049 | -0.001 | [-2.103, 2.010] | -0.044 | 0.965 |
|  | DHL | 1.230 | 0.069 | 0.352 | [1.095, 1.365] | 17.857 | <0.001 |
| Model 6 | Constant | 23.071 | 1.607 | - | [19.919, 26.223] | 14.355 | <0.001 |
|  | Sex | -0.673 | 0.310 | -0.042 | [-1.280, -0.066] | -2.173 | 0.030 |
|  | Age | 0.121 | 0.072 | 0.033 | [-0.019, 0.262] | 1.696 | 0.090 |
|  | Grade level | 0.410 | 0.315 | 0.026 | [-0.207, 1.027] | 1.304 | 0.192 |
|  | Place of birth | 0.033 | 0.315 | 0.002 | [-0.585, 0.651] | 0.105 | 0.916 |
|  | Only-child status | 1.660 | 0.393 | 0.087 | [0.889, 2.432] | 4.220 | <0.001 |
|  | Boarding status | 0.621 | 0.340 | 0.036 | [-0.045, 1.287] | 1.828 | 0.068 |
|  | DHL | -0.346 | 0.024 | -0.299 | [-0.393, -0.299] | -14.498 | <0.001 |
|  | PA | -0.078 | 0.007 | -0.234 | [-0.091, -0.064] | -11.246 | <0.001 |

Note. B = unstandardized regression coefficient; SE = standard error; β = standardized regression coefficient; CI = confidence interval; DHL = digital health literacy; DE = disordered eating; PA = physical activity; PSMU = problematic social media use. Coding: Sex (1 = male, 2 = female); Grade level (1 = middle school, 2 = high school); Place of birth (1 = rural, 2 = urban); Only-child status (1 = yes, 2 = no); Boarding status (1 = yes, 2 = no). Model 1 included all covariates; DHL, PA, and PSMU were sequentially added in Models 2-4. Model 5 used PA as the dependent variable and included the covariates and DHL. Model 6 used PSMU as the dependent variable and included the covariates, DHL, and PA.
